# Feasibility study for estimating optimal substrate parameters for sustainable green roof in Sri Lanka

**DOI:** 10.1007/s10668-022-02837-y

**Published:** 2022-12-21

**Authors:** Shuraik A. Kader, Velibor Spalevic, Branislav Dudic

**Affiliations:** 1 Department of Civil Engineering, Faculty of Engineering, Sri Lanka Institute of Information Technology, Malabe, 10115 Sri Lanka; 2 Biotechnical Faculty, University of Montenegro, Podgorica, 81000 Montenegro; 3 Faculty of Management, Comenius University Bratislava, 82005 Bratislava, Slovakia; 4 Department of Engineering Management in Agribusiness, University Business Academy in Novi Sad, 21000 Novi Sad, Serbia

**Keywords:** Organic wastes, Green roof, Urban ecosystems, Sustainable substrate

## Abstract

In twenty-first century buildings, green roof systems are envisioned as great solution for improving Environmental sustainability in urban ecosystems and it helps to mitigate various health hazards for humans due to climatic pollution. This study determines the feasibility of using five domestic organic wastes, including sawdust, wood bark, biochar, coir, and compost, as sustainable substrates for green roofs as compared to classical Sri Lankan base medium (fertiliser + potting mix) in terms of physicochemical and biological parameters associated with growing mediums. Comprehensive methodologies were devised to determine the thermal conductivity and electric conductivity of growing mediums. According to preliminary experimental results, the most suitable composition for green roof substrates comprised 60% organic waste and 40% base medium. Sawdust growing medium exhibited the highest moisture content and minimum density magnitudes. Biochar substrate was the best performing medium with the highest drought resistance and vegetation growth. The wood bark substrate had the highest thermal resistance. Growing mediums based on compost, sawdust, and coir produced the best results in terms of nitrate, phosphate, pH, and electric conductivity (EC) existence. This study provided a standard set of comprehensive comparison methodologies utilising physicochemical and biological properties required for substrate characterization. The findings of this research work have strong potential in the future to be used in selecting the most suitable lightweight growing medium for a green roof based on stakeholder requirements.

## Introduction

With the exponential population growth and land scarcity, building designers and practitioners have introduced the “green roof” concept to maximise the vegetation in urban areas and to minimise the Urban Heat Island (UHI) effects (Costanzo et al., 2016; Parizotto & Lamberts, 2011; Pianella et al., 2017; K. Vijayaraghavan, 2016). Green roofs also promote effective storm water management with high rates of evapotranspiration (VanWoert et al., 2005). Besides environmental benefits, green roof vegetations enhance the human health by purification of air and improve the social sustainability through the increased coverage of vegetation (Jaffal et al., 2012). Intensive green roofs and extensive green roofs are the two major types of green roofs. Intensive green roofs generally demand high maintenance during their service life (Ampim et al., 2010). Meanwhile, the extensive green roofs that are called “substrate-based green roofs” can be constructed using lower depth substrates. Plants equipped with shallow root systems and with high drought resistance can actively grow on extensive green roofs (Marasco et al., 2014). Most construction stakeholders invest in the instalment of extensive green roofs in urban skyscrapers due to the lower requirement for maintenance and because of their cost-effectiveness. This experimental research study mainly focuses on the effects of various properties for determining substrates using organic wastes for the extensive green roofs in the Sri Lankan context.

Substrate (i.e. growing medium) is the most important component for the enhancement of stability and sustainability of extensive green roofs (Ampim et al., 2010). Green roof substrates are categorised into two main types: mineral substrates and organic substrates (Noya et al., 2017). In terms of mineral substrates, the naturally available topsoil, clay, pumice, sand, and gravel are used as substrates for green roofs due to their great ability to retain moisture (Kader, Jaufer, Shiromi, & Asmath, 2021). However, natural mineral-based growing mediums are hefty in weight, causing additional designs on the roof to maintain the structural stability of the structure (Ampim et al., 2010; Zejak et al., 2022). Lack of nutrients in these substrates persuades stakeholders to make additional investments in artificial fertilisers for efficient plant growth. Thus, use of organic-based eco-friendly growing medium would provide a lightweight substrate with less sulphur content and high water retention to facilitate effective growth of green roof vegetation (Ampim et al., 2010).

Previous research studies have utilised various growing mediums from construction waste materials such as crushed bricks, crushed remains of concrete, formaldehyde resins, and the subsoil (Ampim et al., 2010). Experimental studies were conducted using composite substrates made of USGA sand, dolomite, Turkey litter, and Michigan peat (Noya et al., 2017). One of the major concerns of using mineral substrate as a growing medium for green roofs identified in the study (Soulis, Ntoulas, Nektarios, & Kargas, 2016) was conducted using pumice, zeolite wastes, attapulgite clay, grape marc compost. It was found that due to the heavyweight of these substrates, they provide structural concerns due to the potential cracks in the slabs in skyscrapers that would be caused by the excess weight of the substrate layer.

The heavyweight of the growing medium was the major drawback of using construction waste materials like recycled aggregates. However, these critical issues were not addressed in most of the past studies. The majority of past studies were mainly focused on studying UHI mitigation (Dunnett, 2011), advantages of implementing the green roofs (K. Vijayaraghavan, 2016), and regarding the hydrological performance of green roofs (Galarza-Molina et al., 2017; Stovin, 2010; Xing & Jones, 2021). Some other studies have explored the use of alternative substrate materials for extensive green roofs such as waste silica (Krawczyk, Domagała-Świątkiewicz, Lis-Krzyścin, & Daraż, 2017) and the viability of using recycled aggregates as green roof substrates (Mickovski et al., 2013). These concerns are leading towards finding an alternative substrate material for green roof which could be lightweighted and provide all the subsidiaries required for the plant growth.

Since they are lightweighted, organic wastes-based green roof substrates enhance the structural stability of building structures by preventing the formation of cracks in slabs (Krüger et al., 2019; Rosasco & Perini, 2019; Subaskar, Vidyaratne, & Melagoda, 2017) and they mitigate the additional maintenance and repair costs (Krüger et al., 2019; Sherk et al., 2020). Lightweight substrate with low organic content requires additional fertilisers and water to establish sustainable vegetation (Getter & Rowe, 2006). Furthermore, organic wastes possess high plant nutrient content than the recycled aggregates (Fisgativa et al., 2018; Soobhany, 2019), ideal water retention (S. Kumar et al., 1985), optimum pH, and drought resistance of (Anli et al., 2020) the attributes essential for healthy horticulture. Therefore, the iteration of available organic wastes to find a comprehensive substrate would be a profitable approach in terms of sustainability.

Most global countries are encountering increasing environmental concerns regarding food waste management (Demetriou, 2022; Moshtaghian et al., 2021; Raab, Tolotti, & Wagner, 2021; Xiao & Siu, 2018). Sri Lanka creates 8,000 tonnes of solid waste per day, with around 66% being biodegradable organic waste and nearly 80% being short-term biodegradable food waste (FAO, 2021; Wijerathna, 2021). The generated solid wastes in Sri Lanka are currently being disposed in malpracticeable ways through dumping in water bodies and bare lands (Dharmasiri, 2020), which increased the susceptibility to waterborne diseases like dengue, filaria (Rao et al., 2014), and chronic kidney diseases among the Sri Lankan communities (Rao et al., 2014; Wijkström et al., 2018).

According to Asian Development Bank figures for Sri Lanka, over 4% of the population lives in poverty where approximately 800,000 persons live on less than Rs. 10,000 income per month (FAO, 2021). The alarming increase in financial crisis in Sri Lanka after the post-COVID-19 economic crisis have created an unexpected hike in prices for commercial fertilisers. The current economic crisis has also forced the Sri Lankan urban communities to initiate home gardens to mitigate their ongoing food crisis due to the unavailability of food supplies from rural farmlands. These existing concerns have raised demands in Sri Lanka to formulate alternative lightweight substrate materials for green roofs within low cost and high efficiency, to enhance the urban agriculture for meeting the requirements of 2030 agenda for the sustainable development in developing countries (Desa, 2016).

In terms of waste food disposal, appropriate solutions are being practiced from household levels in countries like Malaysia by validating their feasibility of recycling potential through statistical modelling such as stratified random sampling (Fadhullah et al., 2022). Studies have indicated that the compost derived from domestic sources possesses starch (> 70%) and protein (approximately 14%) in their dry nature (Melikoglu et al., 2013). Plant enzymes like protease, amylase, and amyloglucosidase highly facilitate the breaking down of protein to acquire the required sources for photosynthesis (Simpson et al., 2012). Therefore, using organic compost made of waste meals would be a great solution for providing a nutritious substrate medium for sustainable green roofs in Sri Lanka.

Coir is widely used in substrate layers to enhance the water retention (de Almeida & Colombo, 2021; K Vijayaraghavan & Raja, 2014) similar to sawdust (Depardieu et al., 2016) and wood bark (Yap, Jackson, & Fonteno, 2014). Sawdust is also known for its good nutrient retention ability (Pineda Pineda et al., 2019). Choosing sawdust for green roof substrate would be a wise option to enhance the plant growth within short period of time. Biochar is becoming an increasingly used growing medium due to its remediation capacity of contaminated soils (Parmar et al., 2014). Therefore, this study considered domestically procured organic matters such as coir, biochar, sawdust, wood bark, and compost made of decayable organic matters to implement a feasibility study using the analysis on substrate parameters required for the longevity of green roof vegetation. The findings of our experimental studies based on laboratory and field conditions signify the importance of investigating the viability of using organic wastes as sustainable substrates for green roofs.

It is noteworthy to mention that Sri Lanka contains rich sources of biochar, coir, sawdust, and wood bark (Dharmakeerthi et al., 2012; Gamage et al., 2021). The successful validation from experimental outcomes for using biochar and fellow organic waste materials as green roof substrates would pave way for industrial entrepreneurship in Sri Lanka for substrate production, which could be a sustainable way to resolve the increased national issues related to solid waste management. It would also draw the interest of Sri Lankan construction firms on implementing green roof systems in Sri Lankan urban cities to mitigate the environmental pollution and to fulfil the food requirements, due to the extensive availability of lightweight substrates sources within the country. Similar approach can also be adapted by fellow countries to identify their readily available organic wastes and to test with the recommended parameters for deducing the viability of using those perishable organic materials as successful growing mediums for green roofs.

## Research methodology and materials

### Preliminary study for finding substrate mix ratios

Optimum mix proportions were determined using a field test on buffalo grass species using planting pot growth studies. Base medium was used in this research study as a control specimen, and it was prepared by mixing 90% commercial fertiliser with 10% topsoil since it is a classical Sri Lankan agricultural practice (Dandeniya & Caucci, 2020). A preliminary study was conducted on the selected organic wastes, namely biochar, coir, wood bark, sawdust, and compost (made of animal manure, vegetable remains, and waste meals), to find their corresponding optimum mix proportions with the base medium. The ratios of organic waste to base medium tested in the preliminary study are: 60:40 (w/w%), 70:30 (w/w%), 80:20 (w/w%), and 90:10 (w/w%). Each organic waste species was allocated 60 cylindrical planting pots of 15 cm in height and 10 cm in diameter. Each ratio type had its own three replicates. This experiment was executed at atmospheric temperature and pressure conditions.

Buffalo grass stems measuring 4–6.3 cm were laid in the pots with substrate specimens. These buffalo grass stems were reared from the same buffalo grass breeds to validate that the stems are of similar health conditions and same ages (Dareeju, Meegahage, & Halwatura, 2010). The planted substrate specimens were manually watered and monitored once per week for 8 weeks under Sri Lankan field conditions. The growth of the grass stems was determined by measuring the length from the bottom to the apex (Nagase & Dunnett, 2010). The most suitable mix proportions for the tropical climatic conditions of Sri Lanka were selected for undergoing the successive laboratory experimental analyses following the preliminary study.

### Variables and models

#### Sieve analysis for particle size distribution

The sieve analysis was conducted according to the American Society for Testing and Materials (ASTM) guidelines under C136-01 standards (C136/C136M-14, 2014). Initial weights of substrate specimens were measured and sieve analysis was carried out with 0.30, 0.60, 1.18, 5.00, 10.00, 20.00, 37.50, and 50.00 mm sieves. The Microsoft Excel 2019 spreadsheet was used to calculate the experimental results, such as the weight of the specimen that remained in the sieve and the percentages of passing. The compressibility of a soil mixture is proportional to the amount of fine aggregates (i.e. particle size 2.36 mm) in the substrate (Soane & van Ouwerkerk, 2013). Fine aggregates in the substrate specimens were calculated, and the compressibility of the six substrates was compared.

#### Substrate density and its moisture content

Tray tests were implemented using 25 cm x 25 cm dimensional trays. The test abided by the ASTM 1762–84 standards. Substrate densities (i.e. unit weights) were measured for 2 cm substrate depths for each specimen (Soane & van Ouwerkerk, 2013). To facilitate water discharge in each tray, samples were saturated by pouring with water. Since the selected substrate species are undisturbed soils, indirect approach (Al-Shammary et al., 2018) was used for density calculations. Tray weights W_1_ (in kg) were subtracted from the total weight of the system to determine the net weight of the growing mediums. Then the saturated unit weights of the growing mediums were calculated with Eq. 1.

1 $$\gamma_{st} = \left( {\frac{Ws - W1}{{V1}}} \right) \times \frac{9.81}{{1000}}$$

where W_s_ is the saturated weight of the substrate (including the tray weight) (in kg), V_1_ is the system volume (in m3), and *γ*_st_ is the saturated density of the substrate (in kg/m^3^). Specimens were dried for 24 h at 120 °C in an electrical oven. The dry density of substrates γ_d_ (in kg/m^3^) was determined using Eq. 2.

2 $$\gamma_{{\text{d}}} = \left( {\frac{{W{\text{d}} - W}}{V1}} \right) \times \frac{9.81}{{1000}}$$

*W*_d_ is the weight of the dried specimen (including the tray weight) (in kg), and W is the weight of tray. The relationship between saturated density and dry density is developed using Eqs. 3 and 4 where m_1_ is the moisture content (in %).

3 $$g_{{\text{d}}} = \frac{st}{{1 + m1}}$$

4 $$m1 \, = \frac{st}{{\text{d}}}{-} \, 1$$

#### Drought tolerance

The experiment was set up to find the extent of each substrate’s ability to withstand extreme drought conditions under intense solar light with limited water availability. The results of the experiment helped to deduce the viability of each substrate species in supporting its vegetation during the encounter of an extreme drought. The survival area of buffalo grass is high in a substrate that has less evaporation and high water retention. This metric was used to determine the best drought-resistant substrate. To execute this in situ experiment, six green roof platforms with dimensions 1 × 2 m complying with in situ conditions were constructed by having substrates of biochar, coir wood bark, sawdust, compost, and base medium. Buffalo grasses were installed for 100% coverage in each platform and nursed for a week under the usual nutrition and water supply. By the end of week 1, the water for green roof systems was disconnected, the incidence of sunlight was prevented by covering all six green roof prototypes with opaque polyethene, and the prototypes were allowed for 12 weeks without water and solar light. The dead grass area was calculated per week with 100-cm^2^ square-shaped wooden platforms (Melikoglu et al., 2013) for 84 days. The survival area of vegetation (λ_%_) was calculated by Eq. (5).

5 $$\lambda _{\% } = \frac{{{\text{Platform}}\;{\text{ Area - Dead }}\;{\text{grass}}\;{\text{ area}}}}{{{\text{Platform}}\;{\text{ area }}}} \times 10^{2}$$

#### Growth rate test for specimens

Substrates were installed and green roof prototypes with similar dimensions (i.e. 1 × 2 m platforms) as in the drought tolerance test to determine the ideal substrate that can achieve the highest vegetation cover. A spiderwort species called “*Tradescantia fluminensis*” from the same origin was used in all six prototypes in this growth rate test since *Tradescantia fluminensis* exhibits rapid growth ability even under the availability of low moisture (Hogan & Myerscough, 2017). Each of the six substrate systems was supplied daily with 500 ml of water. The 100 cm^2^ wooden frames were used for measuring vegetation cover. The measurements were undergone on a weekly basis throughout the experimental period of 8 weeks. Vegetation cover (*δ*_*v*%_) in each prototype was calculated per week using Eq. 6.

6 $$\delta_{v\% } = \frac{{{\text{Platform Area}} - {\text{Dead grass area}}}}{{\text{Platform area}}} \times {1}0^{{2}}$$

#### Thermal conductivity test

**Fig. 1:**
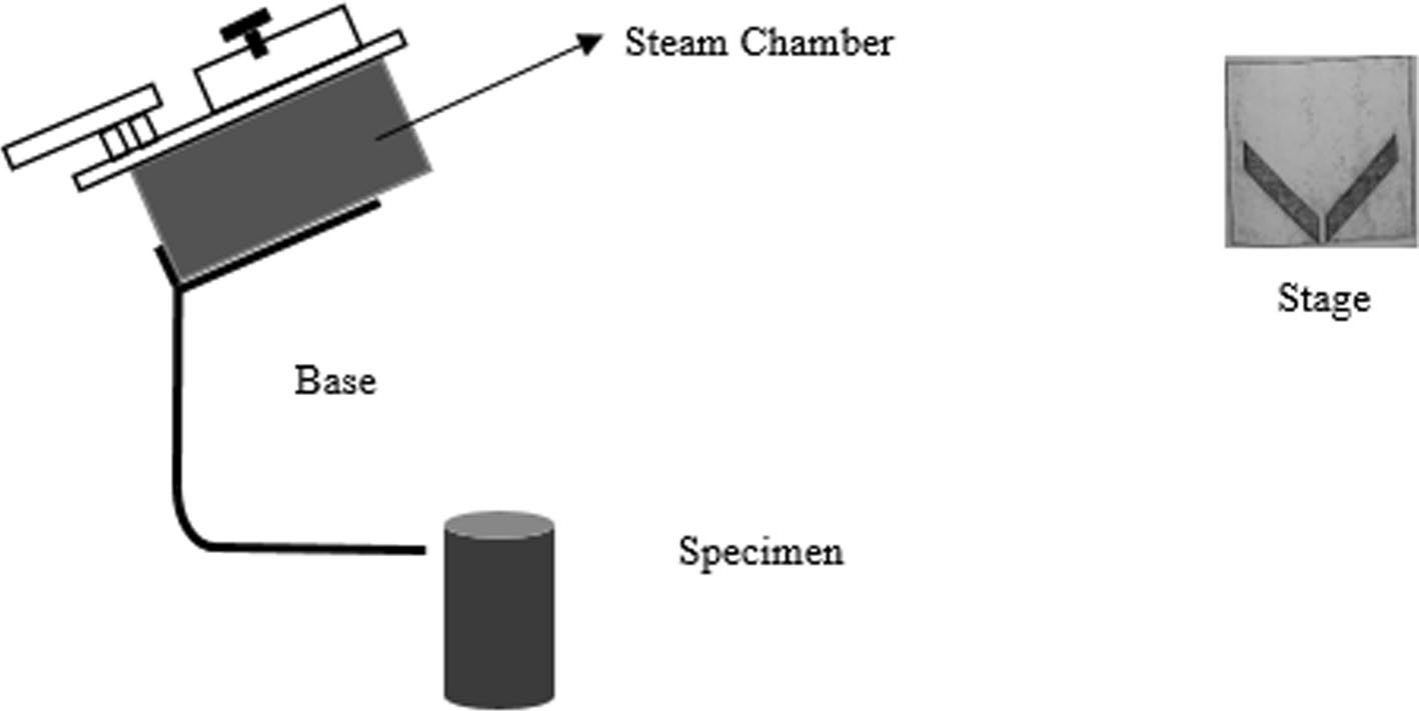
Experimental set-up of thermal conductivity test (Shuraik & Lizny, 2022)

The main objective of this laboratory experiment (as in Fig. 1) is to determine the most thermally resistive substrate among the selected specimens because a highly thermal-resistive substrate will protect the thermal comfort of the building by avoiding the heat conduction from solar radiation on green roofs. Although it is accepted that Lee’s apparatus can determine the thermal conductivity of poor conductors (Kharshiduzzaman, Hossain, Ali, & Ahmed, 2019; Philip & Fagbenle, 2014; Sombatsompop & Wood, 1997), it is not applicable for this test because soil mixtures with organic wastes (i.e. substrate candidates) cannot be moulded into disc shapes due to their heterogeneity and voids. Lee’s apparatus is effective only on uniform specimens. Therefore, the current study used theoretical calculations along with the readings from thermal conductivity apparatus to determine the thermal conductivity of the substrates. Heat conducted by the specimen for a given duration is expressed by Eq. (7).

7 $$\Delta Q \, = \frac{kA\Delta T\Delta t}{h} \times 100$$

ΔQ is the total heat conducted (in W), A is the area of thermal conduction (in cm^2^), *ΔT* is the temperature difference measured (at K), k stands for the thermal conductivity of a given material (in W/m.K), *Δt* is the duration of thermal conduction (in seconds), and h is the thickness of the specimens (in cm). From Eq. 7, the thermal conductivity of a substrate *k* (in cal cm^−1^ s^−1^℃^−1^) is defined:

8 $$k = \frac{\Delta Qh}{{100A\Delta T\Delta t}}$$

Using thermal conductivity computations, thermal resistance was determined with Eq. 9, where R is thermal resistivity in K/W and Δx is the width of the substrate specimen in cm.

9 $$R\Theta = \frac{\Delta x}{{Ak}}$$

Figure 1 shows the experimental set-up devised to find thermal conductivity and thermal resistance by the mentioned mathematical calculations. Specimens were converted into semi-solid mixtures to avoid discrepancies due to voids. Then, the specimens were mounted on the stage and the steam chambers could get heated. When the specimens were observed with the initial melting of water from the system, the time duration was recorded until the next 20 min. The melted specimen was collected in a beaker in 20 min, and the steam chamber was disconnected from the specimen. The collected specimens in the beakers were measured for their weights and corresponding temperatures. The calculated values were used in Eq. 8, and the thermal conductivities were determined. Mean values for thermal conductivity were taken for each substrate from their readings on the original specimen and three replicates. Finally, thermal resistance was determined for the substrate candidates using the available parameters.

#### The pH test for substrates

Optimum pH is vital for green roof plants to prevent leaves from spotting, bronzing, and mineral deficiency (Bailey et al., 2000). In this experiment, the pH test was carried out in accordance with ASTM E70 standards. (Industrial & Chemicals, 2015) in laboratory conditions at a 25℃ temperature.

Substrate solutions were prepared using 1:2 (V/V) (substrate: deionised water) based on ASTM (E70) guidelines. Solutions were transferred to conical flasks and shaken at 200 rpm in a rotary flask shaker for 90 min until the clear solution was observed. 125--mm filter papers were used to transfer the specimen solutions to beakers. Filter papers were enfolded as a floral pattern to increase the contact area with the solution particles and increase the filtration rate. Finally, pH was tested in extracted solutions using a multimeter.

#### Electric conductivity (EC) test of growing mediums

EC is the metric which helps to forecast salinity which is an important parameter in selection of the appropriate green roof substrate (Gougoulias et al., 2013). Excess salinity will cause cytotoxicity in soil due to high concentrations of Na^+^ and Cl^−^ ions that accelerate nutritional imbalance (Isayenkov & Maathuis, 2019) due to exceeding water stress. This would drastically damage the soil health and would hinder plant growth. It is highly advisable to select substrates that consist of salinity within the allowable threshold range.

Saturated media extract (SME) technique is suitable to find the absolute EC of specimens (Huang et al., 2006) but consumes a great deal of time (Kader et al., 2022). Plug sensors methods are not preferred here since they are highly compatible with homogenous solutions (dos Santos Sousa et al., 2021; Visconti & de Paz, 2016). Therefore, a laboratory-oriented method was proposed in basis of pour-through technique (Altland, 2021; Bañón et al., 2021; Palimąka, Pietrzyk, & Sak, 2016) to find the simultaneous EC magnitudes of test specimens.

Solutions prepared for pH tests were used to find EC using a multimeter at room temperature, and some observed that the EC values found were not steady and rapidly fluctuated, corresponding to minor changes in atmospheric temperature. It resulted in various discrepancies to initiate a comparative study. The determined EC magnitudes of specimens were used to construct an EC vs temperature curve to determine EC values at 25 °C, which is the absolute temperature.

#### Mineral contents of substrates

There were three main tests undertaken to find the mineral contents in green roof substrates, namely the total dissolved solids (TDS) test, nitrate test, and phosphate test. The TDS test was carried out using a multimeter in the same solution used to find EC and pH. However, specimens were subjected to spectroscopy using a DR-5000 spectrophotometer to determine their nitrate and phosphate contents.

During the solution preparation for the spectroscopic test for nitrates, 10 ml of sample solutions from each of the six specimens was pipetted into 50-ml volumetric flasks. Then the sulphuric acid (H_2_SO_4_) in 13 N concentration was incorporated with the specimen solutions and the mixture could become a uniform solution by shaking at 180 rpm for 120 min in a rotary shaker. The solution was kept in a cold-water bath at 10 °C and mixed with brucine-sulphanilic acid (C_29_H_33_N_3_O_7_S) to facilitate thermal equilibrium of the resulting solution. The process was halted when colour formation was observed, and the experimental set-up was left undisturbed in atmospheric conditions. Finally, all six specimens were subjected to a spectroscopic test at 410 nm wavelength in the same set of procedures, and their corresponding absorbance data were recorded.

For the calculations, 50 ml of specimen solutions were pipetted into a 500 ml of volumetric flask and then interacted with 3 ml of ascorbic acid (C_6_H_8_O_6_) and 5 ml of ammonium molybdate (NH_4_)_2_MoO_4_). The final substrate solutions were mixed uniformly in a rotary shaker. Afterwards, the solutions were diluted by 80 ml of distilled water and allowed to attain chemical equilibrium to enhance maximum colour formation. This solution was spectroscopically tested at 610 nm, and absorbance data for all specimen solutions were collected.

## Experimental results and discussions

### Preliminary study results

**Fig. 2:**
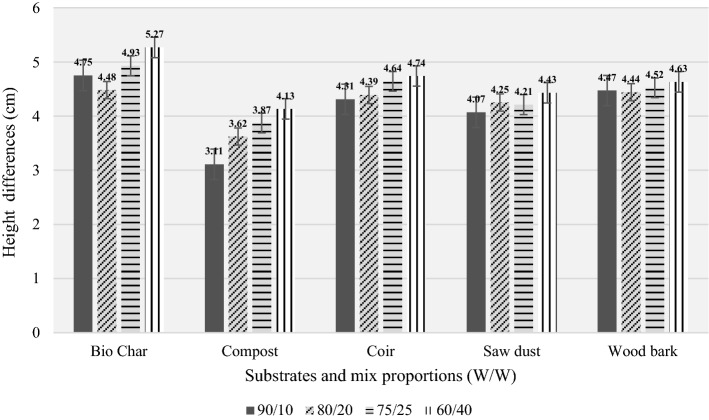
Preliminary study results

Observations have shown that the buffalo grass stems planted in substrate specimens during the preliminary study remained healthy. All stems were alive by the end of the preliminary study duration. It has been indicated that the selected organic waste specimens have good potential to be used as growing mediums. Figure 2 represents the height differences from the bottom to apex in each substrate specimen type by the end of 8 weeks (i.e. after 56 days). Overall results indicate a 60:40 mix proportion as the optimum organic waste-to-base medium ratio for the highest plant growth in each of the candidate specimens for the tropical climate of Sri Lanka.

Biochar supports plant growth more than the other candidates because the maximum growth under each set of ratios is visible in biochar itself. The main reasons for this exceptional behaviour in biochar will be identified in further tests with the optimum mix proportion identified.

Within the 60:40 mix proportion identified, biochar and coir exhibit the highest growth, while compost and sawdust exhibit the least growth. By considering these experimental outcomes, specimens of all organic waste types were prepared at 60:40 mix proportions for these experiments and comparative studies. It is highly recommended for researchers to choose the optimum organic waste to base medium ratio by conducting a similar type of in situ preliminary study in respective to the climatic conditions of their own regions.

### Sieve analysis for particle size distribution

**Fig. 3:**
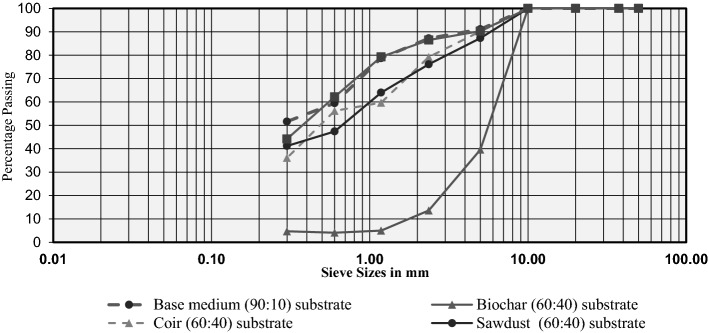
Sieve analysis results

Figure 3 represents the particle size distribution (PSD) curve for passing percentages of aggregates and the respective sieves. Sieves in 5.00–50.00 mm sizes did not retain particles. The fine aggregate percentage is in Table 1 for the comparison of compressive strengths among substrates.

**Table 1:** Fine aggregate percentages in substrate specimens

Name of substrate	Percentage of particles less than 2.36 mm size
Biochar (60:40)	13.6
Sawdust (60:40)	76.1
Coir (60:40)	79.2
Wood bark (60:40)	83.5
Compost (60:40)	86.3
Base medium (90:10)	87.2

The aggregate size of all particles from the produced substrates is less than 10 mm, as shown in Fig. 3. Secondly, during the sieve analysis experiment, it was shown in particle size distribution curve that the dust production was significantly larger in the biochar (60:40). When compared to the other possibilities in Fig. 3, it has resulted in a significant decrease in the proportion of particles retained at sieve size smaller than or equal to 5 mm. This dust formation would also be a potential threat to the industrial applications of biochar substrate in green roofs (Gelardi et al., 2019; Kochanek et al., 2022). In such scenarios, it is highly recommended to lay the substrate layer under a systematic approach rather than spreading as a lump to avoid material wastage as dust.

The durability of a growing medium to be installed on a green roof could be characterised by its compressive strength. The compressive strength of a growth media is related to the fine components that comprise the medium (Das & Sobhan, 2014). When the compressive strength becomes high, it would increase the bearing capacity of soil which is the ability to withstand external loads (Ngo et al., 2020; Ukpata et al., 2012). Based on the results from Table 1, compressibility would be highest in the base medium (90:10) followed by the compost (60:40). The worst compressive strength would be in Biochar (60:40). As a result of its extraordinary compressive strength, base medium (90:10) seems to have the maximum bearing capacity as a green roof substrate to handle external stresses such as plants weight, grass cover, root system, and the associated imposed loads.

### Substrate density and its moisture content

**Fig. 4:**
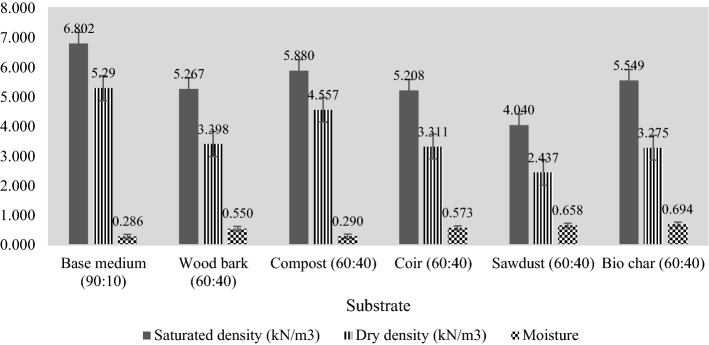
Test results on moisture and density

The calculated unit weight magnitudes and moisture contents for each substrate specimen are described in Fig. 4. The highest saturated density and dry density magnitudes were exhibited by the base medium (90:10), while the minimum saturated and dry densities were observed among the sawdust (60:40). A minimum difference between saturated and dry density values is observed among compost (60:40), which shows that the weight of moisture in compost is comparatively much lower than the weight of organic matter in compost. Base medium and compost consist of significantly high unit weights and low moisture content among the selected substrates. The research findings show that the installation of base medium and compost as green roof substrates would cause challenges to the stability of building slabs due to their heavyweight. Both of these substrate species would incur additional water bills due to the requirement of constant water supply since both of the substrates have less water retention ability, which is evident from moisture content results in Fig. 4.

Sawdust (60:40) demonstrates superior results due to its lightweight properties and better moisture content. The density magnitudes and moisture content of sawdust substrate calculated in this test verify the research findings from the past studies that state Sawdust has less weight and high water retention (Harmayani, 2012; Johansson et al., 2012; Yasin et al., 2020). From this perspective, the final outcome of this experimental study states that sawdust is the better substrate for green roofs in terms of weight concerns and water retention.

### Drought tolerance

**Fig. 5:**
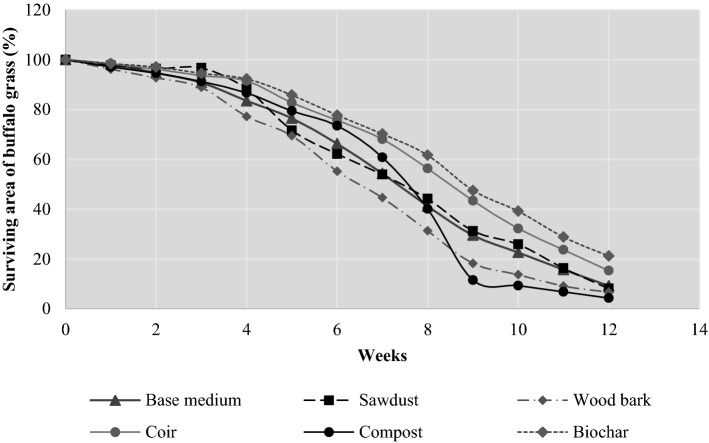
Rate of survival in substrate specimens against the drought conditions

The maximum vegetation cover is observed among biochar (60:40) according to the survival rate chart in Fig. 5. Coir is the second best substrate in this 12-week study to identify the most sustainable substrate under extreme drought conditions.

Compost (60:40) is the least resistive substrate. It is interesting to see that the survival percentage of buffalo grass in compost (60:40) was higher than the percentage of survival in base medium (60:40), wood bark (60:40), and sawdust (60:40) during the first couple of weeks, and this trend continued until the end of week 7. However, the survival rate in compost (60:40) was dramatically reduced in successive weeks and gradually it became the least-friendly substrate for plant survival under drought conditions. The abundant existence of pathogenic microbes in compost (60:40) due to its high organic content from the raw materials of compost substrate would have increased the pathogenic activities, and it would have affected buffalo grass fibrous roots.

Unlike the scenario in compost (60:40), the pathogenic activity in biochar (60:40) was effectively mitigated due to the resistance of biochar against the soil-based pathogens (Bonanomi, Ippolito, & Scala, 2015). In a past study using water partitioning tests, biochar was proven to have the lowest rate of evaporation (Basso et al., 2013; H. Kumar et al., 2020). According to the results from Xie et al. (2013), the addition of biochar improves the germination of seeds due to its high water retention and enhances the unhindered nutrition transport from substrate particles via the fibrous root system of buffalo grass. These distinct properties of biochar would have aided the overlaid buffalo grasses in managing nutrition and water loss under controlled experimental conditions, thereby slowing the rate of vegetation loss. The substantial drought resistive ability of coir (60:40) is due to its pathogen resistivity (Jacoby et al., 2017).

The overall experimental results have shown that biochar (60:40) is the best specimen having the highest drought resistance. The experimental conclusion is supported by a past study conducted on wheat species (i.e. *Tritisivum aestivum*) have verified the strong drought resistance of biochar (Haider et al., 2020). It is also evident from these experimental outcomes that the main factors deciding the drought tolerance ability of a sustainable green roof substrate are the rate of evaporation and pathogen resistance.

### Growth rate of substrate specimens

**Fig. 6:**
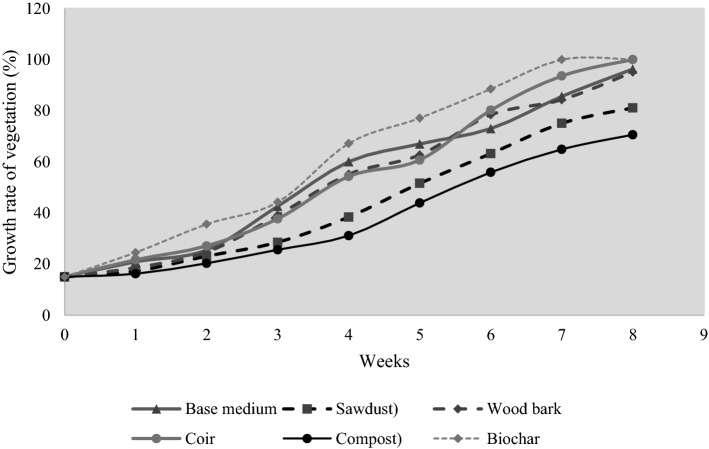
Growth rate test results

Based on the growth test results in Fig. 6, the highest rate of growth is observed in the biochar (60:40). In week 8, both the coir (60:40) and biochar (60:40) substrate green roof prototypes were completed and filled with vegetation cover. Similar to the drought resistance test, compost (60:40) exhibited the least growth. According to the findings from previous research, biochar actively absorbs ammonia compounds consistently and releases them into the soil, which enhances the quality of soil with increased nitrate levels (Busscher et al., 2010; Saarnio et al., 2013) and facilitates sustainable agriculture on green roofs. Since the base medium mixture is a loamy soil, adding biochar has increased the water retention of the biochar (60:40) substrate (Busscher et al., 2010). It would also have facilitated the unhindered photosynthesis of overlaid vegetation (i.e. *Trandescantia fluminensis*) due to the continuous supply of water.

Coir is the second best specimen in terms of supporting plant growth due to its effective air-to-water equilibrium and its good rewetting capacity (Barrett et al., 2016; Blok & Wever, 2005) compared to the other substrate candidates. The minimum vegetation area was observed in compost (60:40) due to the enriched degradation of microorganisms abundant in the embedded animal manure, waste meals, and vegetable remains.

Sawdust (60:40) was a surprise candidate which exhibited the second lowest growth contribution with less favourable outcomes. The main reasons for the least satisfactory outcomes in compost and sawdust are the drawing out of nitrogen compounds from the substrate specimens (S. S. Kumar & Shankar, 2019; Zhang, Liu, Lu, & Zhang, 2020). The biomatter in sawdust quickly decomposes under the exposure to water when the vegetation is watered in experimental activities. This organic degradation uses the available soil nitrogen and fails to supply the nitrogen to the vegetation species (i.e. *Tradescantia fluminensis*). If there are any studies that want to be conducted to enhance the viability of using sawdust as the green roof substrate, then it is highly recommended to find a comprehensive mechanism to mitigate the long-term degradation of sawdust to avoid the loss of substrate fertility.

### Thermal Conductivity test

**Fig. 7:**
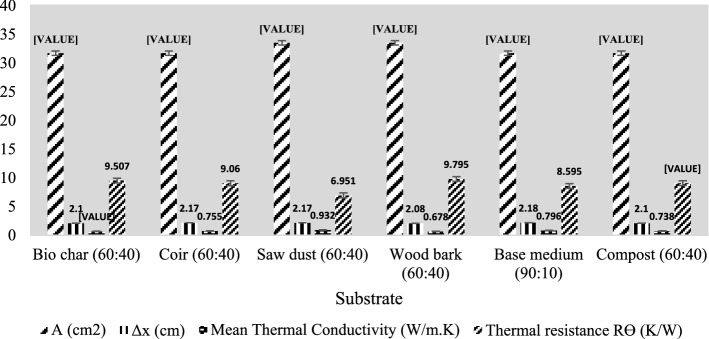
Thermal conductivity results

This thermal conductivity experiment remains a comprehensive attempt to determine the thermal conductivity of green roof substrates using a mathematical approach based on experimental findings, which was recently proven with publication (Shuraik & Lizny, 2022). For analytical calculations, mean values of each test parameter were determined. Figure 7 represents the calculated mean thermal conductivity results for the five substrates and is compared to the base medium. Mean values were considered for thermal conductivity since the results can be influenced by changes in the environmental temperature, by the change in pressure, and due to human errors.

The outcomes of Fig. 7 exhibit thermal conductivity magnitudes of 0.678–0.932 W/m.K. According to the past studies, all the prepared specimen types are sandy loams. The allowable thermal conductivity range for sandy loams according to the past studies is 0.19–1.12 W/m.K. (Abu-Hamdeh & Reeder, 2000). Since the calculated thermal conductivity magnitudes for the selected sandy loam organic substrates are within the allowable range (0.19 < 0.678 < 0.932 < 1.12 W/m.K), it is verified that the experimental procedures are correct. It is known from (Yang et al., 2017) that the thermal conductivity of pure biochar is 1.5 W/m.K and the thermal conductivity value determined in this experiment for biochar (60:40) is 0.693 W/m.K (< 1.5 W/m.K) which is acceptable.

The main goal of the thermal conductivity test is to identify the optimal substrate for green roofs with good thermal resistance to facilitate the thermal comfort of the building. The highest thermal resistance (R) was found in wood bark (60:40), and the lowest magnitude was found in sawdust (60:40). The major reason for the variation of thermal conductivities is the difference in moisture content among substrates. This experimental finding verifies the concept that the thermal conductivity of substrates is directly influenced by their moisture content (Parikh et al., 1979; Riha et al., 1980; Shuraik & Lizny, 2022; Yadav & Saxena, 1973). In terms of the final outcomes, wood bark (60:40) is the most preferred option upon thermal resistance in the growing medium.

It is essential to mention that the calculated values of all the substrates were not compared with past research studies since there were no records available on the thermal conductivity and thermal resistance of the substrates made using the same ingredients and at the same mix proportions. There might be several reasons cited for this drawback of research, such as the complex nature of soil aggregate mixtures and the unpredictability of the behaviour of environmental temperature on soil aggregates.

### The pH test for substrates

**Fig. 8:**
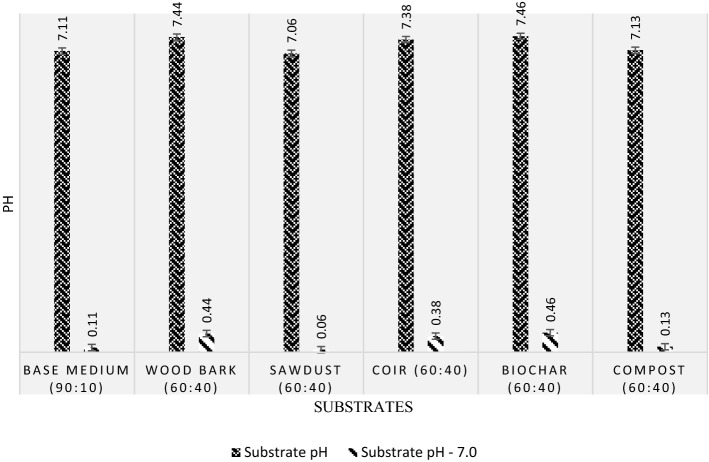
Magnitudes of substrate pH

The pH test results in Fig. 8 show that all the selected growing medium candidates exhibit an alkaline nature since their pH values were greater than 7.0 in this experimental study. Biochar (60:40) exhibits maximum pH (7.46), and the least pH in this study (7.06) is possessed by sawdust (60:40). Raw elements of biochar, coir, and wood bark would have turned more alkaline due to adding the 40% base medium during the substrate preparations. Such drastic alkalinity, on the other hand, is not observed in compost, sawdust, or base medium substrates.

The variation of pH among substrate solutions is due to the existence of carbonyl, nitrile, and phosphoryl groups in different proportions. The intermolecular reaction of these compounds results in the formation of less electronegative resultants (Chhetri et al., 2020). As a result, the number of reactive hydrogens falls. This promotes the formation of more free hydrogen and accelerates the pH of substrates.

The FLL standards recommend a pH of 6.0–8.5 for horticultural vegetation (Eksi & Rowe, 2019), and this requirement is met by the prepared substrate solutions according to Fig. 8. Since sawdust (60:40) has exhibited the closest pH value to 7.0, it is recommended as the most compatible substrate species among the selected contenders, in terms of pH. A substrate with a neutral pH of 7.0 is recommended to create optimum conditions for vegetation in substrate (Kader et al., 2022). In rational perspective, the substrate with a pH magnitude closest to 7.0 was decided to be proposed as the best growing medium for species in terms of pH.

### Electric conductivity (EC) test of growing mediums

**Fig. 9:**
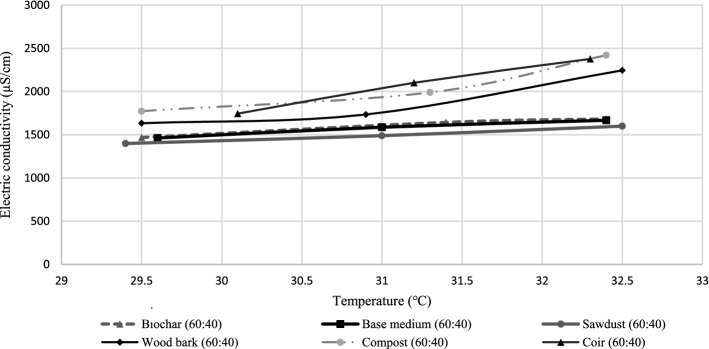
Projected electric conductivity curve

**Fig. 10:**
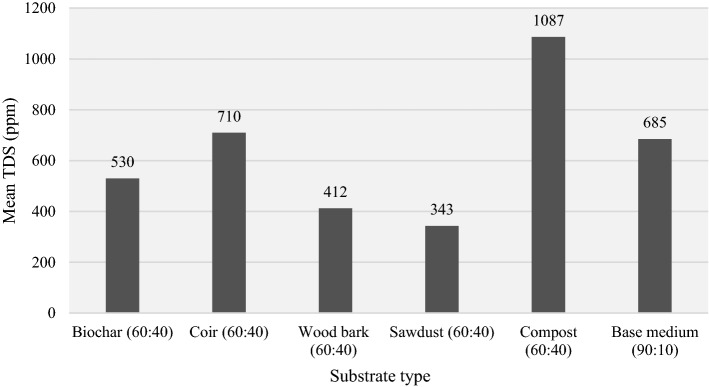
TDS computation on test specimens

**Fig. 11:**
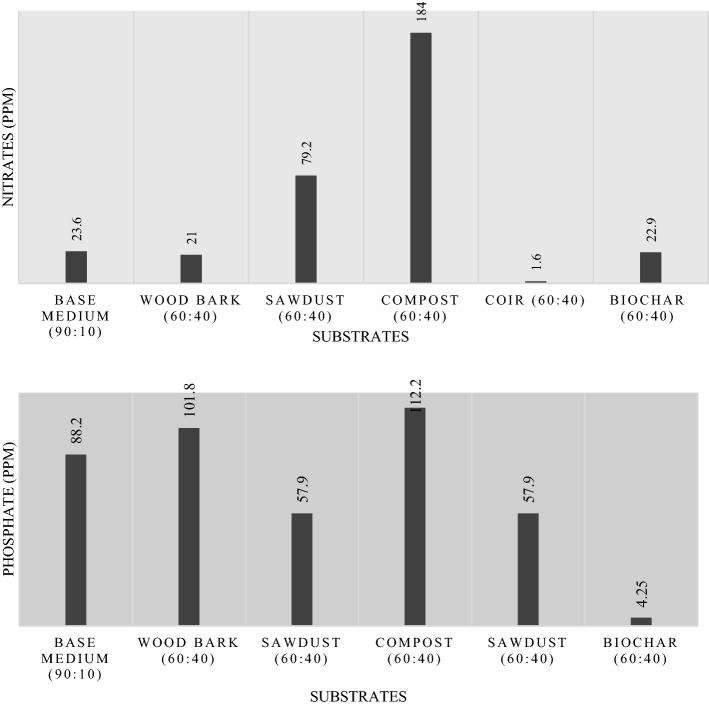
Mean nitrate and phosphate contents of growing mediums

Electric conductivity (EC) values in the first column of Table 2 were obtained for each growing medium to verify the electric conductivity of the substrate medium. According to Fig. 9, the obtained results were calculated for an absolute temperature magnitude of 25 °C using the EC vs temperature graph. The projection of EC into arithmetic equation facilitates the researchers to find EC magnitudes for corresponding substrates at an arbitrary temperature within the experimental range. This approach resolves the inferences from room temperature fluctuations (Kader et al., 2022), atmospheric pressure (Ono & Mibe, 2015), and related environmental conditions (Figs. 10 and 11).

**Table 2:** Rationalised EC test results

Growing medium	EC_1_	Temperature 1	EC_2_	Temperature 2	EC_3_	Temperature 3	Equation of EC curve	Projected EC at 25℃
Base (90:10)	1462	29.6	1587	31	1667	32.4	y = 367.18e^0.0469x^	1186.01
Wood bark (60:40)	1633	29.5	1734	30.9	2245	32.5	y = 66.573e^0.1074x^	975.484
Sawdust (60:40)	1398	29.4	1489	31	1599	32.5	y = 390.74e^0.0433x^	1153.48
Coir (60:40)	1744	30.1	2101	31.2	2377	32.3	y = 25.476e^0.1408x^	860.962
Biochar (60:40)	1468	29.5	1643	31.4	1683	32.4	y = 350.52e^0.0487x^	1184.31
Compost (60:40)	1773	29.5	1989	31.3	2420	32.4	y = 83.257e^0.103x^	1093.27

The objective of an EC experiment is to find the substrate that is equipped with the least salinity, or in other words, the least EC because the lowest salinity would be observed in the substrate with the least EC. Salinity needs to be at its lowest to enhance the highest rate of photosynthesis because the low salinity of soil enhances rich water uptake via xylem tissues (Baum et al., 2000) and prevents the detrimental effects of drought to cell integrity and chlorophyll content (Hussain et al., 2020). Thus, the optimum substrate would consist of the least EC among the selected candidates (Parizotto & Lamberts, 2011).

Base medium (90:10) is the least recommendable substrate according to Table 3 since it possesses the highest EC because higher amounts of nitrates are discharged with runoff water from soil and it mitigates the soil fertility. The photosynthesis potential for plants laid in base medium substrate would be the least due to high loss of minerals from the substrate layer. Furthermore, high salinity of base medium (90:10) and biochar (60:40) would also substantially diminish the phosphorus uptake of green roof plants due to the precipitation of calcium phosphate (Bano & Fatima, 2009). Based on the examined results in Table 2, coir (60:40) possess the least EC and it would facilitate less escape of minerals from the substrate layer (Shrivastava & Kumar, 2015). The overall EC test results recommends coir (60:40) as the best substrate in terms of salinity requirements for green roof substrates since it possesses the lowest EC and would help with better trapping of essential minerals within the substrate layer of green roof.

**Table 3:** Performance ranking of each substrate for each variable

Variable	Biochar	Sawdust	Coir	Wood bark	Compost
Particle size distribution	1	2	3	5	4
Density and moisture content	2	1	4	5	3
Drought resistance	1	3	2	4	5
Growth rate	1	3	2	4	5
Thermal conductivity	2	5	4	1	3
pH	5	1	2	4	3
EC (Salinity)	1	2	5	4	3
TDS	3	5	2	4	1
Nitrates	3	2	5	4	1
Phosphates	5	3	4	2	1

### Mineral contents of substrates

The substrates for green roofs should be rich in nutrients to facilitate long-term nutrient supply. It avoids the additional costs for stakeholders in purchasing fertilisers. The parameters such as total dissolved solids (TDS), nitrates, and phosphates were determined for the selected substrate types to find the optimum substrate in terms of nutrient availability. According to TDS outputs, compost (60:40) consists of high organic and inorganic minerals, and it was closely followed by coir (60:40) in second. At around 347 ppm, sawdust (60:40) has the lowest mineral composition.

The highest amount of mineral content is available in compost (90:10). The second best is coir (60:40). Hence, the least dissolved compositions prevail in sawdust (60:40). TDS magnitudes were measured using three samples (original substrate solution and its two replicates) for each substrate using a multimeter.

The overall consideration of TDS test results along with the pH test outcomes is essential in this experimental study because the substrate gets rich with dissolved minerals under high TDS and the optimum pH (Eridani, Wardhani, & Widianto, 2017; Qi et al., 2009; Rahmanipour et al., 2014). High magnitudes in both TDS and pH hinder the nutrient absorbance rate from soil by root hairs (Guda, 2018). Therefore, in any TDS-based tests, the experiments also incorporate pH analysis to achieve a comprehensive outcome.

Nitrates and ammonium compounds are used by plants for protein synthetization through the production of amino acids. Soil nitrates are essential for plant growth and to maintain chlorophyll health (Sen et al., 2016). The results of the nitrate test recommend compost (60:40) as the optimum substrate in terms of nitrate abundancy.

The main soil phosphates are in orthophosphates, which are the combination of mineral soil and organic content. As in TDS, the increased pH magnitudes disrupt plant roots from the intake of phosphorous. Under the low pH conditions, the orthophosphates react with iron and aluminium to form ferric phosphate (FePO_4_) and aluminium phosphate (AlPO_4_). The pH value of 5.5 to 7.20 is the ideal for optimum P availability (da Silva Cerozi & Fitzsimmons, 2016). Compost is the best growing medium for nutrient availability because it has a pH of 7.13 (< 7.2) and the highest magnitudes of TDS (1087 ppm), nitrates (184 ppm), and phosphates (122 ppm) compared to other substrates.

### Performance analysis of substrates

Table 3 provides a comprehensive performance ranking of each substrate based on tested variables. The physicochemical and biological properties described within the scope of this experimental study have substantially influenced the results exhibited by each substrate candidates. One of the main defect in terms of green roof related research studies are the lack of field experiments since most of the studies are based on laboratories and greenhouses (Gougoulias et al., 2013; Kader et al., 2022). This experimental study has provided a feasible solution for this concern by providing a pioneer experimental framework in this article which incorporates physicochemical and biological parameters with 10 types of laboratory and field experiments.

In this research study, biochar (60:40) exhibited the best characteristics in 4 out of 10 experiments and performed decently in the rest of the tests. Specifically, the performance of biochar in field-oriented tests like the preliminary study experiment, drought tolerance test, and growth rate test is exceptional. Since field condition results are highly preferred in Sri Lankan agriculture more than laboratory outcomes (Shoji et al., 2020), we recommend biochar as the most sustainable growing medium for Sri Lankan climatic context. However, it is best advised for fellow researchers to select the most convenient substrate based on project-specific objectives and the sustainability requirements such as the distinguished climatic conditions of the corresponding region. The overall research findings of this study will be a good guideline for future research studies on finding the sustainable growing mediums for green roofs.

## Conclusions, recommendations, and future research

The optimum growing medium is one of the most important requirements that facilitate sustainable green roofs in the building construction industry. The current experimental research study was designed and systematically conducted to compare the physicochemical and biological characteristics of five soil-organic waste substrates made of sawdust, coir, biochar, compost, and wood bark as the main growing materials. Based on the observations of the preliminary study, 60:40 was selected as the most compatible organic waste/base medium mixing ratio for all five substrate specimens in terms of plant growth stability. Biochar is the substrate with the highest drought resistance and growth potential with ample potential to fulfil the field requirements. The outcomes of this experimental study suggest using a substrate component made of 60% biochar and 40% Sri Lankan traditional base medium as the substrate layer for green roofs in Sri Lanka. Future studies should focus on producing biochar substrates with high nitrates, phosphates, and potassium (NPK) content to reap maximum agricultural benefits because the lack of nutrient content in biochar-based substrate is a major constraint to implement it as a commercial green roof growing medium.

All the organic waste substrates in this study are alkaline. A mathematical approach was utilised to determine the electrical conductivity of substrate specimens through iterating the experimental outcomes at 25 °C temperature by constructing arithmetic equations. Coir (60:40) emerged as the least-saline substrate, which could greatly facilitate the nutrient uptake in plant roots during water seepage. Compost made from vegetable remains, waste meals, and animal manure possesses high nutrients because of its rich amino acid content. However, other experimental results show that compost has a high evaporation rate and it is highly prone to pathogen attacks.

Green roof substrates made from organic waste would provide a more lightweight growing medium than most traditional substrates currently in practise in the Sri Lankan construction industry. Therefore, it is an optimum alternative solution to protect the slabs of green roof buildings from cracks due to substrate weight. It also enhances the soil health with an abundance of nutrients and more water retention. In this research, a method of conducting the preliminary investigation regarding the suitability of using domestically available organic wastes as green roof substrate is comprehensively elaborated with all the required tests, standard guidelines, justifications for the observations, novel methodologies, and the potential outcomes for minimising the environmental impacts to promote sustainability. However, the selection of the best green roof substrate is project specific and often depends on factors such as budget constraints, project specification requirements, and sustainability goals and requirements.

Future research could focus on developing long-term studies on laboratory, greenhouse, and field conditions by incorporating computer algorithms to optimise the best substrate satisfying all the project-specific requirements for reaping the maximum practical outputs in terms of solid waste management and sustainable agricultural practices in urban ecosystems.

## Data Availability

The data sets generated during and/or analysed during the current study are available from the corresponding author on reasonable request.
